# Disrupting the brain to validate hypotheses on the neurobiology of language

**DOI:** 10.3389/fnhum.2013.00148

**Published:** 2013-04-24

**Authors:** Liuba Papeo, Alvaro Pascual-Leone, Alfonso Caramazza

**Affiliations:** ^1^Department of Psychology, Harvard UniversityCambridge, MA, USA; ^2^Center for Mind/Brain Sciences, University of TrentoRovereto, Italy; ^3^Berenson-Allen Center for Noninvasive Brain Stimulation, Beth Israel Deaconess Medical Center, Harvard Medical SchoolBoston, MA, USA; ^4^Institut Guttmann de Neurorehabilitació, Universitat AutonomaBarcelona, Spain

**Keywords:** neuromodulation, action understanding, neuroimaging, cognitive neuropsychology, language semantics

## Abstract

Comprehension of words is an important part of the language faculty, involving the joint activity of frontal and temporo-parietal brain regions. Transcranial Magnetic Stimulation (TMS) enables the controlled perturbation of brain activity, and thus offers a unique tool to test specific predictions about the causal relationship between brain regions and language understanding. This potential has been exploited to better define the role of regions that are classically accepted as part of the language-semantic network. For instance, TMS has contributed to establish the semantic relevance of the left anterior temporal lobe, or to solve the ambiguity between the semantic vs. phonological function assigned to the left inferior frontal gyrus (LIFG). We consider, more closely, the results from studies where the same technique, similar paradigms (lexical-semantic tasks) and materials (words) have been used to assess the relevance of regions outside the classically-defined language-semantic network—i.e., precentral motor regions—for the semantic analysis of words. This research shows that different aspects of the left precentral gyrus (primary motor and premotor sites) are sensitive to the action-non action distinction of words' meanings. However, the behavioral changes due to TMS over these sites are incongruent with what is expected after perturbation of a task-relevant brain region. Thus, the relationship between motor activity and language-semantic behavior remains far from clear. A better understanding of this issue could be guaranteed by investigating functional interactions between motor sites and semantically-relevant regions.

## Introduction

To know a thing is to have information about that thing. To know what “sea” means implies to have information about the appearance, color, texture, taste, temperature, shape, and so on, of that thing. The compositional nature of a concept may be captured by its cortical representation, involving the collective activity of multiple brain regions, each carrying information more or less specific to the various aspects of a concept. One objective of cognitive neuroscience is to define which brain regions are necessary parts of the semantic network, which house core, abstract or general, information about a concept, and which code for specific (e.g., perceptual, functional or motor) aspects.

In word comprehension, an *ad-hoc* distinction can be drawn between *classic* language-processing regions, i.e., brain regions that are generally accepted as part of the language-semantic network, and brain regions that are traditionally regarded as *motor* substrates, and more recently implicated in *higher*-cognitive functions, including language. The recruitment of motor regions, primarily documented with neuroimaging, has greatly impacted the empirical and theoretical work on the nature of conceptual representations and the mechanisms through which the brain implements abstract concepts and symbolic operations.

Here we briefly illustrate cases in which transcranial magnetic stimulation (TMS), has contributed to refining hypotheses about the function of language-related regions, developed in cognitive neuropsychology and neuroimaging research. We then consider how the same methodology has been applied to investigate the nature of language-related motor activity.

## TMS to study language

A TMS pulse adds noise in the neural activity of a relatively focal cortical region (Walsh and Cowey, 2000; Ruzzoli et al., 2010). This perturbation transiently (i.e., with a temporal resolution of a few tens of milliseconds) disrupts the normal ongoing activity in the target region, which results in a behavioral change. This general principle, common to the various TMS protocols (single-pulse, repetitive, paired-pulse, and theta burst stimulation), can inform on whether and at what point in time the target region contributes to a behavior^1^.

The logic underlying the use of TMS to study the neural bases of cognitive functions is analogous to the logic of cognitive neuropsychology. In both cases, we derive conclusions on the brain-behavior relationship based on the effects of “perturbation” on a cognitive system, induced by either stimulation or lesion. In addition, TMS enjoys the advantage of a virtual spatial resolution of a few mm–0.5 cm (Brasil-Neto et al., 1992), as opposed to the widespread lesions of most neurological conditions studied by neuropsychologists. Note, however, that the spatial advantage is only relative, as TMS in humans cannot target spatially specific neural connections. It rather affects a mixture of systems that may interact in producing the final outcome. Moreover, the current induced by TMS can shunt through the corticospinal fluid, reaching locations outside the target region (Wagner et al., 2007). Keeping this in mind, TMS is useful to reveal that one specific region, among the many that show up in neuroimaging scans or that are encompassed by a patient's lesion, is necessary for a complex function, such as language-semantics, *or*—at least—is connected to others that are necessary for that function.

**Figure 1:**
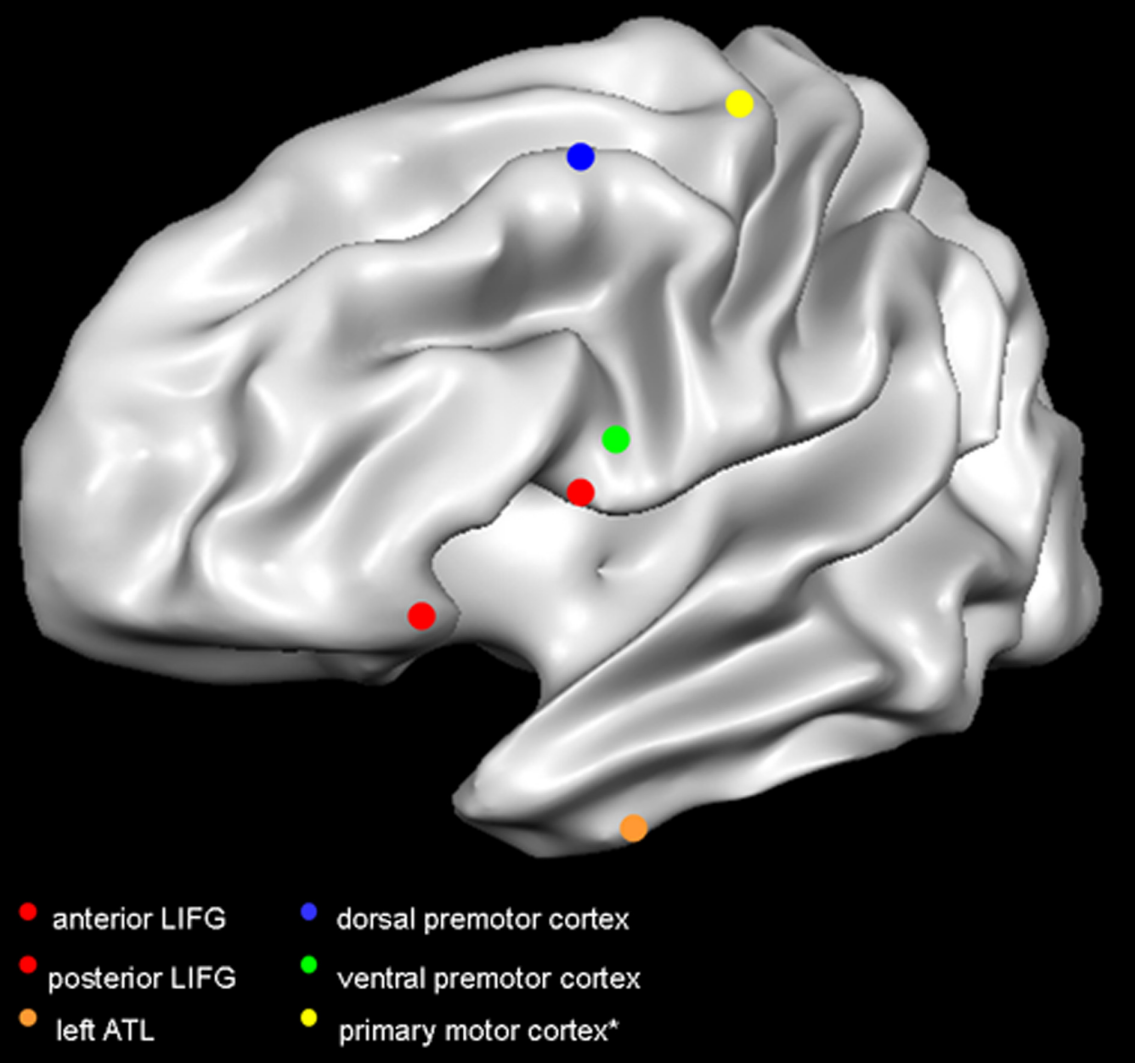
**Target sites in the reviewed studies.** Red dots indicate the TMS targets in Gough et al. (2005); orange dot indicates the target site in Pobric et al. (2007, 2010); blue dot indicates the target in Willems et al. (2011); green dot indicates the target in Cattaneo et al. (2010); yellow dot indicates the primary motor cortex. It is not common practice to report the mean coordinates of the primary motor cortex, as researchers rely on MEP amplitude to target the optimal scalp position. The primary motor cortex is here represented according to the mean coordinates of activity in hand-movement localizer task performed in the fMRI scanner (Papeo et al., 2012). Dots are positioned on a Talairach-normalized “Colin” template, according to the mean coordinates reported in the studies (see Table 1). Coordinates originally reported in Montreal Neurological Institute format, have been converted in Talairach format. Abbreviations: LIFG, left inferior frontal gyrus; ATL, anterior temporal lobe.

As an example, a semantic function of anterior temporal lobes (ATL) was initially developed in the context of neurological studies (Hodges et al., 1992; Mummery et al., 2000; Brambati et al., 2006), but it was inconsistently supported by neuroimaging research, due to methodological limitations only recently overtaken (see e.g., Anzellotti et al., 2011). TMS has contributed to the field, by showing that perturbation of ATL (see Table 1; Figure 1) delayed the performance of healthy individuals on semantic tasks (vs. equally-demanding tasks on numbers), with a greater impact on subordinate-level (*robin*) than basic-level (*bird*) objects (Pobric et al., 2007), a phenomenon sometimes observed in patients with semantic dementia. Later, Pobric et al. (2010) showed that TMS to ATL delayed participants' naming of objects, regardless of their category (living and non-living), while TMS over the left inferior parietal lobe (IPL) only affected naming of manipulable non-living objects. Converging with neuropsychological (Hodges et al., 1992; Mummery et al., 2000; Brambati et al., 2006), and neuroimaging results (e.g., Mummery et al., 1996; Kellenbach et al., 2005; Anzellotti et al., 2011), it is likely that ATL perturbation was directly responsible for the semantic task-specific impairment reported by Pobric et al. (2007). At the same time, we are more cautious in assuming a category-general function of ATL (Pobric et al., 2010). This skepticism, motivated by reports of category-specific effects in neuroimaging (Anzellotti et al., 2011) and neuropsychological studies on ATL (Brambati et al., 2006; Bi et al., 2011), takes into account the caveat that TMS can directly affect only the lateral aspects of the cortex. Thereby, its behavioral consequences might not capture the function of medial/ventral aspects, within the reach of other methodologies.

**Table 1:** **Mean-group coordinates (*x*, *y*, and *z*) of cortical regions targeted with TMS in the main studies discussed in this review**.

	**Cortical region**	***x***	***y***	***z***
**“CLASSIC” LANGUAGE-PROCESSING REGIONS**				
Gough et al. (2005)	Anterior inferior frontal gyrus	−52	34	−6
Pobric et al. (2007)	Posterior inferior frontal gyrus	−52	16	8
Pobric et al. (2010)	Anterior temporal lobe	−53	4	−32
**FRONTO-CENTRAL MOTOR SITES**				
Cattaneo et al. (2010)^*^	Ventral premotor cortex	−48.9	4.6	20
Willems et al. (2011)	Dorsal premotor cortex	−35	−1	53

All regions are in the left hemisphere. Coordinates of primary motor cortex are not reported, as researchers commonly rely on MEP amplitude to target the optimal scalp position for stimulation of this site. Where not otherwise specified, coordinated are in Montreal Neurological Institute (MNI) format.

* Coordinates are in Talairach format.

The spatial resolution of TMS, combined with proper control conditions, can help to distinguish between very close and densely connected sites. For instance, researchers have extensively debated whether the left inferior frontal gyrus (LIFG), recruited in both speech production and comprehension, served one general function or was a functionally heterogeneous region. By delivering TMS to either the anterior (aLIFG) or the posterior aspect (pLIFG), during both a semantic and a phonological task, Gough et al. (2005) found slower responses to the semantic task after TMS to aLIFG, and to the phonological task after TMS to pLIFG (Table 1; Figure 1). This double dissociation provided compelling evidence that LIFG is in effect a functionally heterogeneous region.

The use of TMS to establish brain-behavior causal relationships extends to the investigation of many language functions. For instance, the general involvement of the left frontal lobe in verb processing, suggested by neuropsychological and neurophysiological studies, could be circumscribed to the anterior midfrontal gyrus (isolated from its posterior part and from the Broca's area) in the TMS work of Shapiro et al. (2001) and Cappelletti et al. (2008). Likewise, being well-known that the left temporal lobe is implicated in semantics, TMS research is now contributing to assign more specific functions to specific sub-portions of this large part of the brain (Whitney et al., 2011; Schuhmann et al., 2012). Also taking advantage of experimental paradigms (e.g., based on RTs) that cannot always be used with neurological patients, TMS research can replicate observations from cognitive neuropsychology, with a greater spatial characterization of behavioral “symptoms.” In the next section, we review and discuss how this potential has been exploited to investigate the nature of precentral motor activity in language understanding.

## TMS outside the *classic* word-semantic network

Reports of language-induced activity in precentral motor regions have given new impetus to the debate on the constituents of conceptual representations (see Mahon and Caramazza, 2008; Binder and Desai, 2011). Concepts may be stored in the form of abstract, modality-independent representations, or symbols, within dedicated cerebral structures, abstracted away from the systems for action, and perception (Fodor, 1983; Pylyshyn, 1984; Shallice, 1988; Caramazza et al., 1990). By contrast to this *cognitivist* account, the notion of *embodiment* characterizes the view that the sensory-motor information, acquired and used to interact in the environment, constitutes the mental representation of that entity. On this view, conceptual processes rely on the sensory and motor structures, carrying out the *internal simulation* of perceptual or motor aspects of the concept (Allport, 1985; Barsalou, 1999; Jeannerod, 2001; Gallese and Lakoff, 2005).

No theory of concepts denies that physical experience is an important aspect in the acquisition of conceptual knowledge, and that sensory and motor information can be involved in conceptual processes. What makes the embodied theory a true alternative to the cognitivist theory is the specific stance that concepts can be reduced to sensory and motor information, namely that activity in *low-level* structures for action and perception exhaustively represents concepts.

A major research effort has been directed to evaluate the “strong” prediction of the embodied hypothesis that understanding actions recruits the whole stream for action execution, up (or down) to the level of the primary motor cortex (M1; Jeannerod, 2001; Pulvermüller, 2005), and the “weaker” prediction that the recruitment of motor regions is more general that the specification of a motor program, entailing representations at the level of premotor cortex (Gallese et al., 1996).

TMS is particularly well-suited to assess these predictions. Delivered over M1, TMS can reach the cortical representation of body parts with a spatial resolution as specific as the level of individual muscles (e.g., first dorsal interosseus, opponens pollicis, abductor digiti minimi of the hand, and so on). A TMS pulse, with intensity above the individual motor threshold (or suprathreshold)^2^, activates the underlying neural population, resulting in a twitch in the peripheral muscles responding to the stimulated area. The amplitude of the twitch, recorded in the form of motor evoked potentials (MEPs), provides a direct measure of corticospinal excitability^3^. This procedure offers the opportunity to test the “strong” prediction that identical substrates (i.e., specific hand-muscles) are recruited when physically grasping and when understanding the word “grasping” but not, for instance, the word “biting.” At the same time, it makes it possible assessing behavioral changes caused by TMS perturbation during a cognitive task. TMS to non-primary motor sites (i.e., premotor cortices) does not elicit measurable MEPs, but it still perturbs the underlying activity and thus allows inferences based on the evaluation of behavioral changes.

In the following sections, we review: (1) studies in which TMS to M1 has been used to measure cortico-spinal excitability and to assess the effect of M1 perturbation on linguistic tasks, and (2) studies with TMS over premotor sites to assess changes in participants' language behavior. This set of studies is now large enough to advance hypotheses on the contribution of motor regions to language understanding.

### TMS over the primary motor cortex

Oliveri et al. (2004) carried out the first TMS study to measure cortico-spinal excitability while participants processed action- and non-action-related words. The authors asked whether the suggested implication of motor regions in verb processing (Bak et al., 2001) reflected a grammatical class effect, or the semantic distinction between nouns and verbs, frequently denoting objects and actions, respectively.

Participants were instructed to generate aloud morphological transformations of visually presented nouns (singular or plural) and verbs (third person singular or plural). Using paired-pulse TMS over the left M1, where a suprathreshold TMS pulse is delivered immediately (10 ms) after a subthreshold conditioning pulse that has the function to pre-activate the target site (Kujirai et al., 1993), increased MEPs were registered from the right hand for action nouns and verbs, relative to non-action nouns and verbs.

These results provided indication that the motor cortex is sensitive to the action vs. non-action distinction of word meanings. The authors, however, did not rush to the conclusion that a causal involvement of the motor system in word processing had been proven; they rather emphasized how it could not be clarified whether motor activity concurrent with word processing was necessary for action-word processing, or reflected epiphenomenal spreading activation from the retrieved concept.

Pulvermüller (2005) argued that, in order for motor activity to be regarded as a component of word understanding, it should: (1) be somatotopic, respecting the bodily effector involved in the implied-language action; (2) occur as early as lexical-semantic access (i.e., ~200 ms); (3) occur automatically following word presentation, regardless of task demands, and (4) its perturbation should result in a change of language performance.

Pulvermüller et al. (2005) tested these predictions in a study where participants performed a lexical decision task (i.e., deciding whether a letter string is a word) with words related to arm- and leg-actions (*grasp* vs*. kick*). Subthreshold single-pulse TMS was applied 150 ms after word-onset, to modulate one of the following sites: arm representation in the left or in the right M1 (arm-M1) and leg representation in the left or in the right M1 (leg-M1). As a consequence of stimulation, participants' decision times were faster to arm-words (vs. leg-words) after TMS to the left arm-M1 and faster to leg-words (vs. arm-words) after TMS to the left leg-M1.

**Figure 2:**
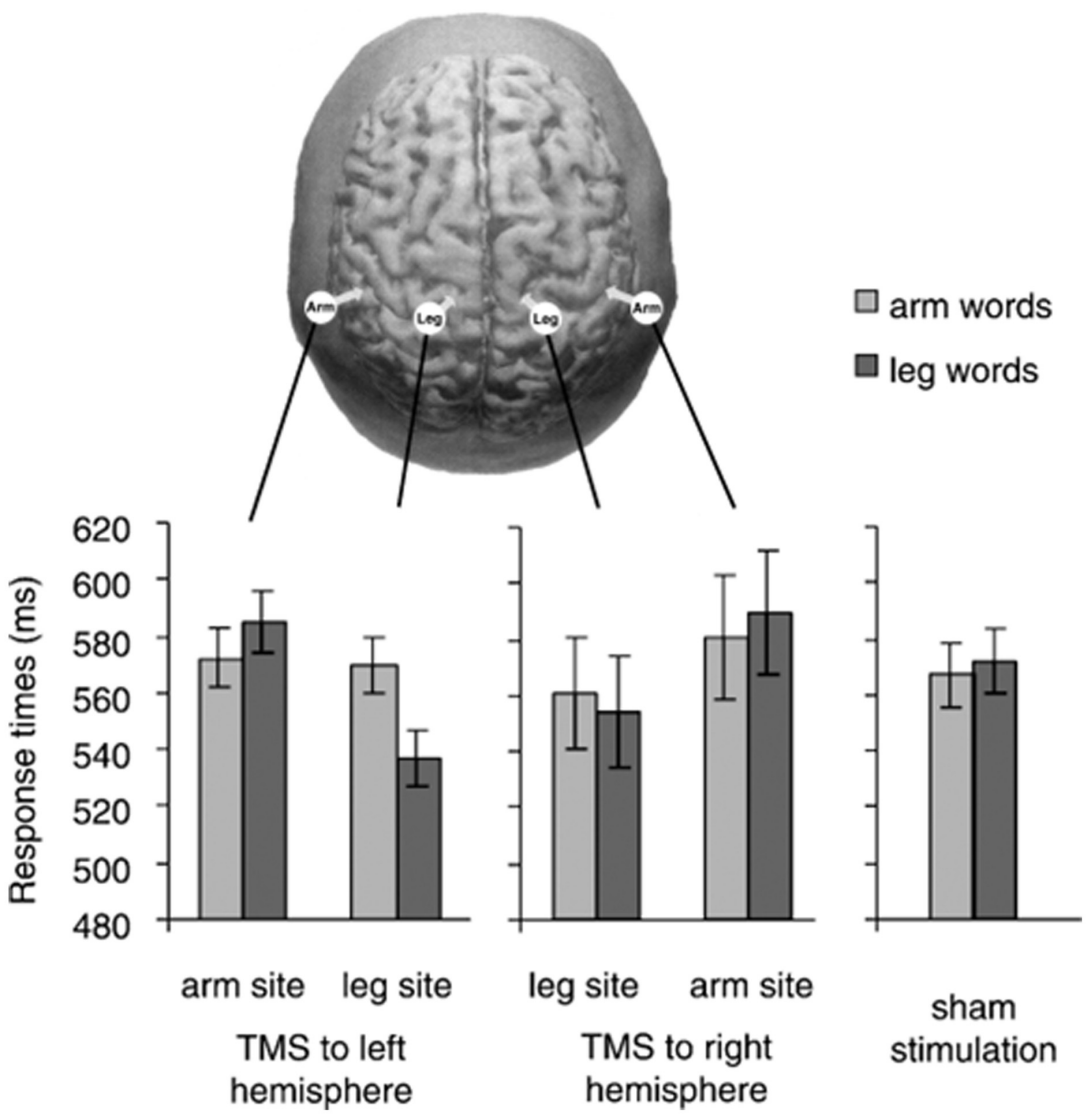
**In the top, the stimulation sites in Pulvermüller et al. (2005) are depicted.** The graphs show response times to arm words and leg words in five TMS conditions (TMS to arm-M1 and leg-M1 of the right and left hemisphere, and sham stimulation). We notice that response times to arm words remained quite unchanged across the three critical conditions (TMS to the left arm-site, to the left leg-site and sham stimulation). With permission from (Pulvermüller et al., 2005).

A tricky aspect of these results is that reaction times (RTs) to arm-words did not seem to differ across conditions with TMS to arm-M1, TMS to leg-M1, and sham-TMS (i.e., baseline condition; see Figure 2); that is, RTs to arm-words remained unchanged irrespective of whether TMS was delivered or not to either M1 site. The pattern of results was thus driven by variation in the performance with leg-words, visibly faster during leg-M1 stimulation than during arm-M1 stimulation. Taking advantage of this one data point, the authors concluded that a subthreshold TMS pulse applied to a region responsible for the semantic processing of words facilitated the upcoming word processing, just like a prime stimulus facilitates the processing of a semantically-related target word.

Assuming that the effect for leg-words was reliable and the analogy with semantic priming properly captures the effect of a subthreshold pulse on semantic processing, these results do not yet clarify the role of M1 in word understanding. In fact, it is equally possible that the activation of leg-M1 was directly involved in lexical decision, or that the “subliminal” stimulation of the leg site, encoded in conceptual regions, *pre-activated* the concept “leg” and thus facilitated the processing of the semantically congruent words.

Other studies used suprathreshold stimulation to elicit MEPs and therefore measure motor activity in language tasks. Buccino et al. (2005) applied single-pulse TMS to the left hand-M1 and leg-M1 and measured MEPs from hand- and foot-muscles, respectively, in correspondence with the acoustic presentation of verbs describing hand-actions (*he took the cup*), foot-actions (*he kicked the ball*), or abstract verbs (*he loved his wife*). Decreased MEPs were recorded from hand muscle after hand-action verbs (vs. foot- and abstract-verbs), and from foot muscle after foot-verbs (vs. hand- and abstract-verbs).

Later, Glenberg et al. (2008) applied suprathreshold TMS to the left hand-M1, while participants performed a semantic-plausibility judgment task on sentences describing physical transfer (*you give the papers to Marco*), abstract transfer (*you delegate the responsibilities to Anna*), or no-transfer (*you read the papers with Marco*). The authors found greater MEPs for both abstract- and concrete-transfer sentences relative to no-transfer items. This facilitation, however, was only found when concrete- and abstract-transfer items were compared, as a single condition, with the no-transfer items; the effect did not reach significance for either transfer-type sentence, when analyzed separately (*p*-values: 0.08 and 0.09 for concrete and abstract items vs. no-transfer items, respectively).

Strikingly, despite the similarity of procedures in Buccino et al. and in Glenberg et al., the two studies reported language-related motor interference and a trend toward facilitation, respectively. Motor facilitation, as reported by Glenberg et al., appears the most reliable result in the current literature (Oliveri et al., 2004; Tomasino et al., 2008; Papeo et al., 2009, 2011a). However, the lack of difference between concrete and abstract language in that study is hard to reconcile with the other TMS (and neuroimaging) studies, where abstract items have been used as the control condition to highlight action word-related motor activity. The authors did not explain how abstract relations can be delegated to motor information to an extent that is not distinguishable from concrete interactions.

Overall, the TMS results discussed so far show a certain variability at least in terms of direction of language-related motor effect (decreased vs. increased activity), and verbal materials associated with the effect (concrete-action vs. concrete + abstract language). This variability could extend to the types of language tasks that elicit motor activity. For instance, Papeo et al. (2009) found that MEPs increased for action-words when participants were instructed to think about the motor components of word-stimuli (semantic task), but not when the access to meaning was only incidental (i.e., in syllable counting). We note that not all studies reporting language-related motor facilitation involved explicit instructions to attend the motor components of words' meanings. Hypothetically, however, stimulation of M1 with its tangible consequence (i.e., the twitch) might act as a cue that activates the motor components associated with a word meaning.

So far, evidence has been provided that M1 activity changes when words with motor components are processed. However, to argue for a causal role of this activity, one should be able to show some sort of quantitative relation between changes in motor activity and semantic performance. While such result has not yet been reported, the behavioral consequences of TMS perturbation could help to approach this question. It is therefore surprising that many studies in the field restricted the data report to the physiological effect of TMS (increased/decreased MEPs), leaving aside its on-line behavioral effect (Oliveri et al., 2004; Buccino et al., 2005; Glenberg et al., 2008).

Studies comparing participants' performance (RTs and accuracy) with and without TMS to M1 gave conflicting results. While the study by Pulvermüller et al. (2005) found partial and unclear behavioral facilitation (see our discussion above), two studies by Papeo et al. (2009, 2011a) found increased M1 activity associated with action-related words, with no indication of action category-specific effect at behavioral level. Another study by Lo Gerfo et al. (2008) reported that, relative to the baseline condition (no TMS), participants were slower in morphological transformation of action words after prolonged exposure to repetitive TMS over the left M1(*offline* protocol). While this protocol may have greater interference strength relative to single-pulse TMS, it increasingly runs the risk of inducing widespread changes in neural activity at long-distant sites connected to the stimulated one (Chouinard et al., 2003; Kobayashi and Pascual-Leone, 2003; Huang et al., 2005). In the absence of any other evidence, the cautious interpretation of Lo Gerfo et al.'s unique observation is, as the authors themselves pointed out, that M1 enjoys connections with semantically-relevant regions. We will later return to this discussion.

### TMS over the premotor cortices

Advocates of the “weaker” embodied hypothesis might argue that perturbation of M1 yields no behavioral change, because semantically-relevant information is contained at the level of premotor cortex, particularly in the ventral aspect of the precentral gyrus (e.g., Gallese et al., 1996; Damasio et al., 2004; Rizzolatti and Craighero, 2004; Martin, 2007). On this view, M1 activity would simply reflect downstream effects of premotor activation.

Cattaneo et al. (2010) used *state-dependent* TMS over the premotor cortex and assessed its effect on processing tool-nouns, a category of words whose meaning is associated with a manipulation program. State-dependent TMS rests upon the principle that physiological effects of TMS result from the interaction between the input-stimulus applied and the initial state of the target region (i.e., its level of activity). The initial state of a brain region, defined as the susceptibility of that region to be activated, can be influenced by any external or internal input, including task demand, experimental setting, individuals' expectations, and psychological state (Silvanto and Pascual-Leone, 2008).

In state-dependent TMS, as used in Cattaneo et al. (2010), the initial state of the target site was modulated behaviorally, through priming. The priming effect (i.e., the facilitation of processing a target-stimulus appearing after a perceptually or conceptually related prime-stimulus; Neely, 1977), is thought to reflect pre-activation or change in tuning of the neural population responsive to the “primed” features (Desimone, 1996; Wiggs and Martin, 1998; Grill-Spector et al., 2006). In a region that contains neurons responsive to a given target-category, a TMS pulse delivered immediately after the prime-stimulus facilitates responses to the target-stimulus when this is unprimed (i.e., preceded by an unrelated prime) relative to when it is primed. One interpretation of this phenomenon is that the firing rate of neurons in the stimulated region increases more, before reaching the ceiling, when the neurons are not pre-activated than when they have been pre-activated by the prime (Silvanto and Pascual-Leone, 2008).

In each participant, Cattaneo et al. (2010) targeted the left ventral premotor cortex (vPMC) as the experimental site and the left dorsal premotor cortex (dPMC) as the control site. Having the word “tool” as prime and nouns of tool-exemplars as targets, they found that categorical decisions (i.e., deciding whether the target was an exemplar of the tool-category) to unprimed targets (tool-names preceded by the unrelated prime “animal”) were faster with state-dependent TMS to vPMC, relative to the conditions with TMS to dPMC and no-TMS. RTs to the primed target (tool-names preceded by the prime “tool”) did not differ across TMS conditions; that is, the priming effect was abolished with TMS to vPMC. The priming effect was never affected by TMS, when prime-target pairs belonged to the “animal” category (control condition).

The category-specific effect in Cattaneo et al. rests upon the assumption that the firing rate of “tool-responsive” neurons in vPMC reached ceiling when the prime “tool” was presented. A parametric variation of the semantic distance of the prime from the tool-concept could prove this assumption true, by showing that the closer the semantic relation of the prime with “tool,” the weaker the facilitation of processing target-tools after state-dependent TMS. Leaving aside this methodological issue, the results by Cattaneo et al. do not clarify what kind of information is represented in vPMC (e.g., biological motion performed by the tool-user or tool motion), but they do provide indication that that brain site contains information, specific to the processing of tool-nouns.

The “virtual lesion” approach could extend those results, revealing selective TMS interference with the semantic processing of tool-nouns. A similar approach has been implemented in Willems et al. (2011) to investigate the role of the dorsal aspect of the premotor cortex (dPMC) in word processing. Participants performed a lexical decision task involving manual-action verbs, non-manual-action verbs (abstract) and legal pseudowords, after exposure to continuous theta burst stimulation (TBS). This protocol affects the excitability of neurons in the motor cortex in the direction of long-term (i.e., up to 1 h) inhibition (Huang et al., 2005).

Based on their own fMRI results (Willems et al., 2010), the authors selected the left dPMC as the target, and the right dPMC as the control site. Decision times to manual-action verbs were faster after TBS over left dPMC, than after TBS over right dPMC; RTs to abstract verbs did not differ with left and right TBS.

Although most theoretical and empirical reports implicate left vPMC in action-related conceptual processing, evidence also exists for semantic category-specific responses in dPMC (Beilock et al., 2008; Postle et al., 2008). Bringing support to the latter position, Willems et al.'s results appear, at first sight, in conflict with those of Cattaneo et al. (2010), where left dPMC was the control site and its stimulation led to no behavioral effect. Recall, however, that in Cattaneo et al. stimuli were nouns, while in Willems et al. they were verbs and, when nouns were used, no effect was found in left dPMC (Table 1; Figure 1).

One possibility is that the left dPMC is recruited when processing verbs and the left vPMC is specific to the processing of nouns. Tool nouns and action verbs carry different types of action-related information: one gross distinction is that, in the case of nouns, action information relates to a specific context (i.e., the specific tool), in the case of verbs, it relates to a specific movement or motor program (e.g., grasping) that applies to several contexts. Conjecturally, the one-to-one vs. one-to-many ratio between the verbal label and the implied motor context could capture the difference between tool-nouns and manual-action verbs and underlie a functional segregation within the precentral gyrus^4^.

One serious issue raised by Willems et al.'s results concerns the direction of the effect: behavioral improvement as opposed to the behavioral impairment that is expected as a consequence of the inhibitory effect of continuous TBS (Huang et al., 2005). If the metaphor of TMS as “virtual lesion” stands, Willems et al.'s pattern is reminiscent of the *paradoxical facilitation* of performance reported in the brain-lesion literature (Kapur, 1996), and interpreted as evidence for a competing/inhibitory function of the lesioned site relative to the assessed behavior. In this perspective, it is entirely possible that the physiological response of dPMC—even if inhibitory—contributes to some aspect of lexical performance. Certainly, the violation of the expected TBS-induced disruption of behavior solicits caution in interpreting Willems et al.'s findings as conclusive demonstration of brain-behavior causality.

## What conclusions can we reach from TMS research?

Studies with TMS to the left M1 have convincingly shown a temporal association between motor activity and variations in word semantics (i.e., action vs. non-action). Moreover, the behavioral effects of TMS perturbation have shown that different aspects of the precentral gyrus are sensitive to the action-non action distinction of words' meanings. However, a selective disadvantage for action-word processing was only reported by Lo Gerfo et al. (2008) with TMS over M1. It is hard to believe that M1 supports a semantic function independently of premotor regions, to which it is strongly connected: ultimately, activity in M1 is the outcome of higher-level premotor activity (Civardi et al., 2001; Gerschlager et al., 2001; Koch et al., 2007). The problem is that, when TMS perturbation was applied directly over the premotor cortex, unexpected facilitation of performance with action-words was found (Willems et al., 2011). So inconsistent effects can be hardly taken as evidence for a direct role of precentral motor sites in the promotion of word encoding; they rather evoke interpretations based on not-yet-clear (inhibitory/competing or excitatory) interactions between the stimulated sites and semantically-relevant regions.

This skepticism is further motivated by results from cognitive neuropsychology. Cross-talk between neuropsychological and TMS research is crucial to evaluate the hypothesis that the local target of stimulation is directly responsible for a given behavioral change (e.g., slower RTs). Although premotor regions are often encompassed by brain lesions, deficits in word understanding are consistently associated with damage to left frontal and temporal regions, but not to motor and premotor sites (Papeo et al., 2011b; Arévalo et al., 2012; Kemmerer et al., 2012). Furthermore, detailed analyses of patients' behavioral profiles have documented spared understanding of action-words in cases of impaired praxis (i.e., impaired ability to execute the actions implied by words) and *vice versa* [Negri et al., 2007; Papeo et al., 2011b; Papeo and Rumiati, 2012; see discussions in Mahon and Caramazza (2005), Papeo and Hochmann (2012)].

On one hand, language can elicit precentral motor activity; on the other hand, an individual is still able to understand an action verb after damage to the system for action production. Then, if motor activity reflects the processing (or *simulation*) of the motor aspects of words' meanings, such activity would be *redundant* to semantic processes, which are held elsewhere in the brain and are on their own sufficient to understand words. Alternatively, information carried by motor activity could complement word processing by serving to ground aspects of conceptual representations in the immediate context in which these are retrieved [see the “grounding by interaction” account in Mahon and Caramazza (2008)]. This interpretation does not necessarily predict that perturbation/damage to motor regions must result in a general impairment of word understanding; while it leaves it open a possibility for future TMS and patients' studies to capture more specific behavioral aspects (e.g., context-specific characterizations or *senses* of a concept) that could be directly dependent on motor activity.

Finally, we have pointed out how the behavioral effects of TMS over the motor sites could imply connectivity between those sites and the fronto-temporal language-semantic network. Besides showing over again that motor regions do respond to words, advances in the field could be made by studying how different word-responsive regions (e.g., motor precentral and associative temporal) interact in terms of functional and effective connectivity. TMS protocols (e.g., dual-site paired-pulse) also combined with neuroimaging methodology, have proven successful to study cortical interactions in *lower-level* functions (e.g., motor control) and could now contribute to this enterprise in the domain of *higher* conceptual tasks.

### Conflict of interest statement

The authors declare that the research was conducted in the absence of any commercial or financial relationships that could be construed as a potential conflict of interest.

## Footnotes

^1^For a description of the physics underlying the interference of TMS pulses with electric brain activity, we refer to a number of papers, which have also the merit of highlighting a number of “unknowns” of this technique (Barker et al., 1985; Pascual-Leone et al., 1999, 2000; Walsh and Cowey, 2000; Wagner et al., 2007). The handbooks by Pascual-Leone et al. (2002); Walsh and Cowey (2000), and Wassermann et al. (2008) are recommended for the interested parties.

^2^Individual threshold is statistically defined as the lowest stimulation intensity of the primary motor cortex that produces motor evoked potentials (MEPs) of 50 μV or more, in the peripheral muscle responding to the stimulated area, in at least 50% of the applied pulses (Rossini et al., 1994). This measure is often taken as a general indication of the individual cortical excitability.

^3^Corticospinal excitability directly reflects the state of M1, but it also provides an indication of activity in high-order premotor regions, which send extensive connections to M1 and to motorneurons in the spinal cord (Luppino et al., 1994; Geyer et al., 1996; Dum and Strick, 2005).

^4^This distinction is possibly compatible with the motor function of the two sites, whereby the vPMC contributes more to object grasping (a fundamental step in manipulation), while dPMC would maintain the selection of the appropriate response, among many, to a cue (see Chouinard and Paus, 2010).
